# Impact of the Narcotics Information Management System on Opioid Use Among Outpatients With Musculoskeletal and Connective Tissue Disorders: Quasi-Experimental Study Using Interrupted Time Series

**DOI:** 10.2196/47130

**Published:** 2024-02-21

**Authors:** Iyn-Hyang Lee, So Young Kim, Susin Park, Jae Gon Ryu, Nam Kyung Je

**Affiliations:** 1 College of Pharmacy Yeungnam University Gyeongsan Republic of Korea; 2 Department of Pharmacy Kosin University Gospel Hospital Busan Republic of Korea; 3 College of Pharmacy Woosuk University Wanju Republic of Korea; 4 Department of Pharmacy Sungkyunkwan University Samsung Changwon Hospital Changwon Republic of Korea; 5 College of Pharmacy Pusan National University Busan Republic of Korea; 6 Research Institute for Drug Development Pusan National University Busan Republic of Korea

**Keywords:** addiction, chronic noncancer pain, monitoring, mortality rate, Narcotics Information Management System, narcotics, NIMS, opioid misuse, opioid prescription, opioid, overdose, prevention, terminal pain, time series, web-based system

## Abstract

**Background:**

Opioids have traditionally been used to manage acute or terminal pain. However, their prolonged use has the potential for abuse, misuse, and addiction. South Korea introduced a new health care IT system named the Narcotics Information Management System (NIMS) with the objective of managing all aspects of opioid use, including manufacturing, distribution, sales, disposal, etc.

**Objective:**

This study aimed to assess the impact of NIMS on opioid use.

**Methods:**

We conducted an analysis using national claims data from 45,582 patients diagnosed with musculoskeletal and connective tissue disorders between 2016 and 2020. Our approach included using an interrupted time-series analysis and constructing segmented regression models. Within these models, we considered the primary intervention to be the implementation of NIMS, while we treated the COVID-19 outbreak as the secondary event. To comprehensively assess inappropriate opioid use, we examined 4 key indicators, as established in previous studies: (1) the proportion of patients on high-dose opioid treatment, (2) the proportion of patients receiving opioid prescriptions from multiple providers, (3) the overlap rate of opioid prescriptions per patient, and (4) the naloxone use rate among opioid users.

**Results:**

During the study period, there was a general trend of increasing opioid use. After the implementation of NIMS, significant increases were observed in the trend of the proportion of patients on high-dose opioid treatment (coefficient=0.0271; *P*=.01) and in the level of the proportion of patients receiving opioid prescriptions from multiple providers (coefficient=0.6252; *P*=.004). An abrupt decline was seen in the level of the naloxone use rate among opioid users (coefficient=–0.2968; *P*=.04). While these changes were statistically significant, their clinical significance appears to be minor. No significant changes were observed after both the implementation of NIMS and the COVID-19 outbreak.

**Conclusions:**

This study suggests that, in its current form, the NIMS may not have brought significant improvements to the identified indicators of opioid overuse and misuse. Additionally, the COVID-19 outbreak exhibited no significant influence on opioid use patterns. The absence of real-time monitoring feature within the NIMS could be a key contributing factor. Further exploration and enhancements are needed to maximize the NIMS’ impact on curbing inappropriate opioid use.

## Introduction

Opioids have traditionally been used to manage acute or terminal pain [1-3]. However, their prolonged use has generated controversy due to the potential risk for abuse, overdose, and addiction [4], leading to negative outcomes and excessive mortality rates [3,5]. Previously confined to cancer-related pain management before the 1980s [6], opioids have gradually expanded their application to noncancer pain management, resulting in an increased incidence of opioid use disorders [7]. The overprescription of opioids has even steered individuals toward illicit substances such as heroin [8]. In 2018, opioid overdoses were responsible for over 60,000 deaths in the United States, making them the leading cause of drug-related deaths [9,10]. This worrisome trend has transcended national borders, manifesting as a significant global public health challenge [11-15]. An ecological study conducted in Spain corroborated this concern, demonstrating a direct correlation between greater prescribed opioid drug availability and opioid-related mortality [16].

The use of opioids in South Korea was lower compared to the United States and Europe, according to the International Narcotics Control Board’s report [17]. However, in recent years, there has been a rapid increase in the prescription rate of opioids [18]. For instance, from 2002 to 2015, the number of patients using opioids chronically for more than 90 days increased 6-9 times [19]. To address this issue, the Ministry of Food and Drug Safety (MFDS) implemented the Narcotics Information Management System (NIMS) on May 18, 2018 [20,21]. Before the NIMS, health care institutions relied on manual record-keeping to manage the use and inventory of opioids [22]. The NIMS is a web-based system that tracks patient information and inventory using a serial number assigned to each unit (bottle and carton) of opioid [22]. The system stores and monitors information at all times, making it easier to track and manage narcotics. The introduction of NIMS has transformed the monitoring of drug handling from a random management process to a selective on-site monitoring system by analyzing drug handling information. The MFDS aims to establish a safer narcotics management network through real-time tracking of narcotic handling history [21,22].

To date, there are no published studies that have directly evaluated the impact of NIMS on opioid use by comparing prescription patterns before and after the installation of the system. Therefore, we aimed to examine whether the NIMS led to any improvement in indicators related to inappropriate opioid use using an interrupted time-series (ITS) analysis. The study period coincided with the outbreak of COVID-19, and the analysis also sought to assess its effect on opioid use.

## Methods

### Data Sources

The analysis was performed using National Health Insurance (NHI) claims data between 2016 and 2020. For research purposes, the Health Insurance Review and Assessment Service (HIRA) annually provides patient sample data (HIRA–National Patient Sample [HIRA-NPS]). HIRA-NPS is derived from health insurance claim data submitted by medical institutions for reimbursement [23]. HIRA provides a sample representing 3% of the Korean population for 2016, 2017, and 2018 and a sample representing 2% of the Korean population for 2019 and 2020.

### Study Design

To assess the impact of NIMS on opioid use, a segmented ITS analysis was performed. The data were aggregated monthly over the 5-year study period. As per the recommended practice, the ITS analysis required observations of at least 12 months before and after the intervention [24]. The NIMS was introduced on May 18, 2018, and the analysis period covered 28 months before the intervention and 32 months after. The study period also included the COVID-19 outbreak, which was treated as a second interruption to control its influence on opioid use. The second interruption occurred in February 2020 in South Korea.

### Study Population

**Figure 1:**
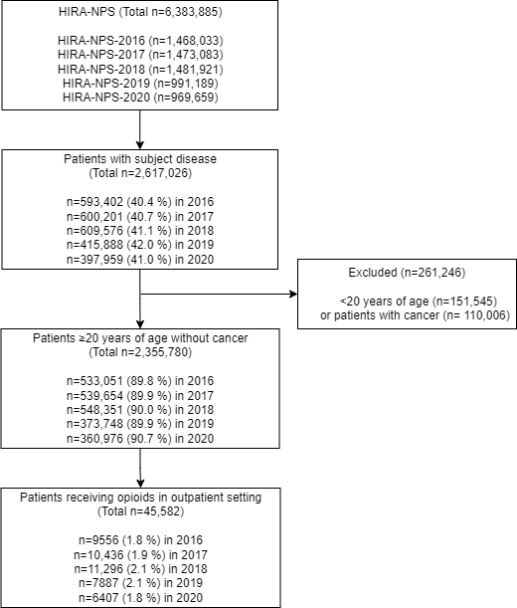
Flowchart of study population selection. The subject diseases are arthropathies, dorsopathies, soft tissue disorders, and other disorders of the musculoskeletal system and connective tissue. HIRA-NPS: Health Insurance Review and Assessment Service–National Patient Sample.

The study included patients with disorders of the musculoskeletal system and connective tissue [25], identified using the *International Classification of Diseases, Tenth Revision* (ICD-10) codes. The subject diseases are arthropathies (eg, osteoarthritis), dorsopathies (eg, spondylosis), soft tissue disorders (eg, fibromyalgia), and others (eg, postprocedural musculoskeletal disorders). Patients younger than 20 years and those with a cancer diagnosis were excluded. Outpatients who received opioid prescriptions were selected as target patients (Figure 1). The patient population was further categorized by age groups (20-39, 40-59, 60-74, and over 75 years) and the types of insurance (NHI and Medical Aid [MedAid] or Patriots and Veterans Insurance Plan [PVI]). NHI covers most of the study population, while MedAid includes individuals with low income. PVI is a special health program for patriots and veterans who have served in the military or government agencies. While PVI is distinct from NHI and MedAid in terms of its target population, it is grouped together with patients with MedAid insurance in our study due to their similar patterns of drug use [26].

### Study Drugs

The study aimed to analyze the use of outpatient opioids in oral and patch formulations. The following opioid medications were included in the analysis: buprenorphine transdermal, codeine oral, dihydrocodeine oral, fentanyl transdermal, hydrocodone oral, hydromorphone oral, morphine oral, oxycodone oral, and tapentadol oral formulations (Multimedia Appendix 1). However, sublingual tablets, buccal tablets, and nasal sprays were excluded from the analysis as they are primarily used for breakthrough pain in patients with cancer, in accordance with the Korean reimbursement criteria [27].

### Ethical Considerations

The study protocol was approved by the institutional review board of Pusan National University, Busan, South Korea (PNU IRB/2022_27_HR).

### Outcome Measures

#### Overview

Throughout the study period, we assessed inappropriate opioid use monthly using 4 key indicators, each carefully selected to encompass different dimensions of inappropriate opioid use based on previous research in this area [28-33]. These indicators included (1) the proportion of patients on high-dose opioid treatment, (2) the proportion of patients receiving opioid prescriptions from multiple providers, (3) the overlap rate of opioid prescriptions per patient, and (4) the naloxone use rate among opioid users.

#### The Proportion of Patients on High-Dose Opioid Treatment

The proportion of patients on high-dose opioid treatment refers to the percentage of patients prescribed high-dose opioid treatments out of all patients included. High-dose opioid treatment was determined by assessing their daily morphine milligram equivalents (MME), a standardized measure of opioid potency that allows for comparing and aggregating each patient’s opioid consumption. For our study’s purposes, a daily dose of 100 MME or higher was considered high-dose opioid treatment [28-30]. Such treatment has been associated with elevated rates of mental health issues, including substance use disorders, and increased health care service use when compared to cases of no opioid use or low-dose opioid treatment [30].

#### The Proportion of Patients Receiving Opioid Prescriptions From Multiple Providers

The proportion of patients receiving opioid prescriptions from multiple providers was calculated to evaluate the dispersion of patient care within the outpatient setting [31]. This computation encompassed patients obtaining prescriptions from 2 or more providers, irrespective of the provider’s institutional affiliation. This was accomplished by summing up the number of providers who issued opioid prescriptions to each patient monthly. The practice of opioid prescribing by multiple providers is widespread and has been linked to elevated rates of hospital admissions directly associated with opioid use [31].

#### The Overlap Rate of Opioid Prescriptions Per Patient

The ratio of overlapping narcotic prescriptions is a measure of the extent to which the opioid prescriptions of study drugs overlap for each patient. It is calculated as the ratio of the overlapping prescription period to the total prescription period of the study drug per patient. Overlapping opioid prescriptions stand as one of the indicators signifying potential opioid misuse [32].

#### The Naloxone Use Rate Among Opioid Users

The percentage of patients prescribed and administered naloxone among opioid users each month is calculated as a proxy for opioid overdose incidents. Naloxone is a widely used medication for reversing the effects of opioid overdose [33], and the proportion of patients receiving naloxone reflects the frequency of opioid overdose or toxicity incidents.

### Statistical Analysis

Descriptive statistics for all variables were presented. The time-series data of outcomes were examined graphically and used to establish a segmented regression model to assess the statistical significance of the effect of NIMS, which was investigated by analyzing the change in opioid use using 4 outcomes [34]. The time-series data of these outcomes were analyzed over 60 months, from January 2016 to December 2020, with the NIMS intervention occurring in May 2018 (29th month). The model to be tested in this study is as follows:


Yt = β0 + β1 × timet + β2 × NIMSt + β3 × time after NIMSt + β4 × COVIDt + β5 × time after COVIDt + εt

where *Y_t_* is the outcome of interest in month *t*; time is a continuous variable, indicating time in months at time *t* from the start of the observation period; *NIMS* is an indicator for time *t* occurring before (NIMS=0) or after (NIMS=1) the NIMS, which was implemented at month 29 in the series; and *time after NIMS* is a continuous variable counting the number of months after the NIMS at time *t*, coded 0 before the NIMS and (time-28) after the NIMS. *β_0_* estimates the baseline level of the outcome at time zero; *β_1_* estimates the baseline trend before the NIMS; *β_2_* estimates the level change at the month of NIMS introduction; *β_3_* estimates the change in the trend after the NIMS, compared with the monthly trend before the NIMS; *β_4_* estimates the level change at the month of the COVID-19 outbreak at month 50 in the series; and *β_5_* estimates the change in the trend after the COVID-19, compared with the monthly trend before the COVID-19 outbreak.

The sum of *β_1_* and *β_3_* is the postintervention slope. As can be seen from the model, each interval is represented by a level and a trend. The change in the level of the outcome after the intervention indicates the immediate effect of the intervention. The change in trend is defined as the change in slope in the interval after the intervention compared to the interval before the intervention [34]. The Durbin-Watson test was used to assess the serial correlation of error terms and estimate the regression coefficients with either an ordinary least squares or a first-order autocorrelation maximum likelihood estimate, depending on the significance of serial correlations [35]. Residual analyses based on autocorrelation plots and partial autocorrelation plots were carried out to review the goodness-of-fit of the model. Statistical analysis was performed using R software (version 4.1.1; R Foundation for Statistical Computing), and when the *P* value was <.05, it was considered statistically significant.

## Results

### Characteristics of Study Population

A total of 45,582 patients received outpatient opioid prescriptions, and the most common subject disorders were dorsopathies, followed by arthropathies and soft tissue disorders. The study patients were predominantly female (27,756/45,582, 60.9%), with the largest age group being 60-74 years (15,752/45,582, 34.6%). The percentage of MedAid beneficiaries among the study patients ranged from 8.8% (2189/24,924) to 10.7% (539/5016), which was higher than the general population (which is approximately 3%). Some fluctuations were observed in sex, age distribution, and insurance type between the 3 time periods (Table 1).

**Table 1:** Characteristics of the study population and overall changes in 4 outcome variables from January 2016 to December 2020 (N=45,582).

Characteristics			Total (60 months; N=45,582)		Pre-NIMS^a^ (28 months; January 2016-April 2018; n=24,924)			Post-NIMS Pre–COVID-19 (21 months; May 2018-January 2020; n=15,642)				Post–COVID-19 (11 months; February 2020-December 2020; n=5016)
**Sex, n (%)**												
	Male	17,826 (39.1)		9591 (38.5)			6125 (39.2)				2110 (42.1)	
	Female	27,756 (60.9)		15,333 (61.5)			9517 (60.8)				2906 (57.9)	
**Age group (years), n (%)**												
	20-39	5706 (12.5)		3236 (13)			1951 (12.5)				519 (10.3)	
	40-59	15,079 (33.1)		8401 (33.7)			5148 (32.9)				1530 (30.5)	
	60-74	15,752 (34.6)		8518 (34.2)			5397 (34.5)				1837 (36.6)	
	≥75	9045 (19.8)		4769 (19.1)			3146 (20.1)				1130 (22.5)	
**Insurance type, n (%)**												
	NHI^b^	41,452 (90.9)		22,735 (91.2)			14,240 (91)				4477 (89.3)	
	MedAid^c^ or PVI^d^	4130 (9.1)		2189 (8.8)			1402 (9)				539 (10.7)	
**Morbidity, n (%)**												
	Arthropathies	11,902 (26.1)		6533 (26.2)			4092 (26.2)				1277 (25.5)	
	Dorsopathies	21,234 (46.6)		11,588 (46.5)			7283 (46.6)				2363 (47.1)	
	Soft tissue disorders	11,908 (26.1)		6534 (26.2)			4086 (26.1)				1288 (25.7)	
	Others^e^	538 (1.2)		269 (1.1)			181 (1.2)				88 (1.8)	
**Outcome variables, mean (SD)**												
	Proportion of patients on high-dose opioid treatment	N/A^f^				0.75 (0.22)			1.25 (0.27)	1.89 (0.30)		
	Proportion of patients receiving opioid prescriptions from multiple providers	N/A				0.69 (0.27)			1.02 (0.27)	1.37 (0.37)		
	Overlap rate of opioid prescriptions per patient	N/A				1.95 (0.42)			2.17 (0.20)	2.30 (0.30)		
	Naloxone use rate among opioid users	N/A				0.57 (0.28)			0.51 (0.16)	0.45 (0.26)		

^a^NIMS: Narcotics Information Management System.

^b^NHI: National Health Insurance.

^c^MedAid: Medical Aid.

^d^PVI: Patriots and Veterans Insurance.

^e^Other disorders of the musculoskeletal system and connective tissue.

^f^N/A: not applicable.

### The Proportion of Patients on High-Dose Opioid Treatment

The proportion of patients on high-dose opioid treatment was the lowest in the pre-NIMS period (187/24,924, 0.75%), followed by the post-NIMS pre–COVID-19 period (196/15,642, 1.25%), and then the highest in the post–COVID-19 period (95/5016, 1.89%; Table 1).

**Figure 2:**
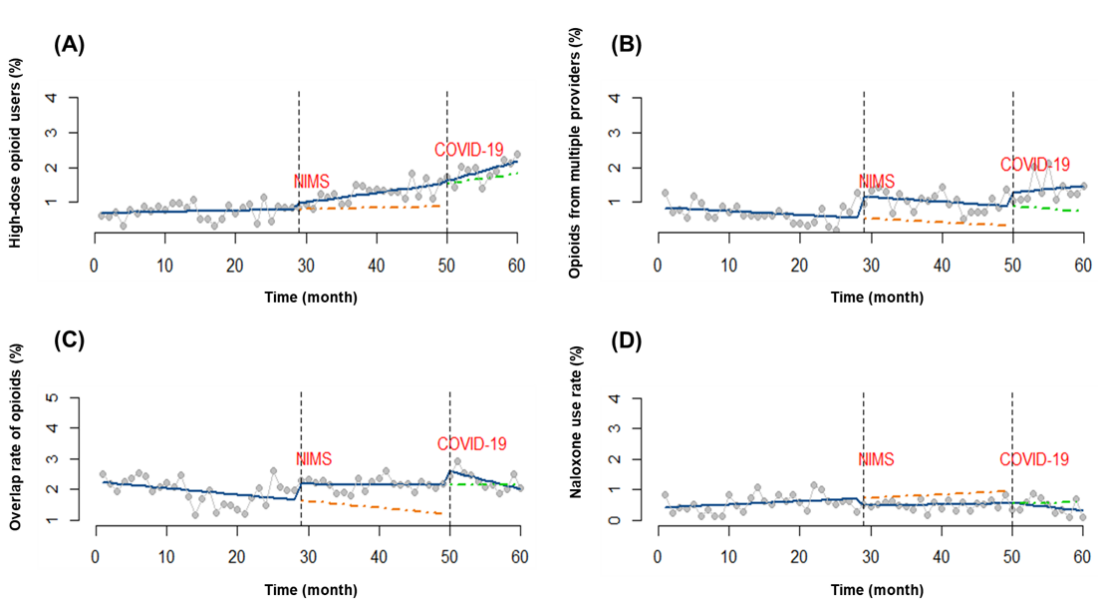
Impact of Narcotics Information Management System (NIMS) and COVID-19 on 4 outcome variables from January 2016 to December 2020: interrupted time-series analysis with observed and predicted regression lines for the (A) proportion of patients on high-dose opioid treatment; (B) proportion of patients receiving opioid prescriptions from multiple providers; (C) overlap rate of opioid prescriptions per patient; and (D) naloxone use rate among opioid users. The first interruption was held in May 2018 and the second was held in February 2020. The blue solid line represents the actual data, while the orange and green dot-dash lines represent the predicted data after NIMS and the COVID-19 outbreak, respectively.

The trend showed a significant increase of 0.0271 from 0.0040 at baseline to 0.0311 (*P*=.01) after the implementation of NIMS, with no significant change in the level. The COVID-19 outbreak had little impact on both the level and trend of the percentage of patients receiving high-dose opioid treatment. The regression results are shown in Table 2 and Figure 2A.

**Table 2:** Modeling interrupted time series to evaluate the impact of the Narcotics Information Management System (NIMS) and COVID-19 on opioid use among outpatients with musculoskeletal and connective tissue disorders in South Korea.

Outcome variables and estimator (AR^a^)		Segmented regression coefficient	*P* value	D-W^b^	
**Proportion of patients on high-dose opioid treatment**					1.9426
	Intercept (β_0_)	0.7947	<.001		
	Baseline trend (β_1_)	0.0040	.48		
	Level change after NIMS (β_2_)	0.1743	.20		
	Trend change after NIMS (β_3_)	0.0271	.01		
	Level change after COVID-19 (β_4_)	0.0484	.78		
	Trend change after COVID-19 (β_5_)	0.0309	.19		
**Proportion of patients receiving opioid prescriptions from multiple providers**					2.0825
	Intercept (β_0_)	0.8141	<.001		
	Baseline trend (β_1_)	–0.0076	.31		
	Level change after NIMS (β_2_)	0.6252	.004		
	Trend change after NIMS (β_3_)	–0.0067	.59		
	Level change after COVID-19 (β_4_)	0.3969	.09		
	Trend change after COVID-19 (β_5_)	0.0323	.28		
**Overlap rate of opioid prescriptions per patient**					2.0046
	Intercept (β_0_)	1.4828	<.001		
	Baseline trend (β_1_)	–0.0113	.16		
	Level change after NIMS (β_2_)	0.3349	.08		
	Trend change after NIMS (β_3_)	0.0101	.46		
	Level change after COVID-19 (β_4_)	0.3709	.12		
	Trend change after COVID-19 (β_5_)	–0.0442	.16		
**Naloxone use rate among opioid users**					1.9854
	Intercept (β_0_)	0.3685	.001		
	Baseline trend (β_1_)	0.0156	.01		
	Level change after NIMS (β_2_)	–0.2968	.04		
	Trend change after NIMS (β_3_)	–0.0117	.26		
	Level change after COVID-19 (β_4_)	0.0652	.72		
	Trend change after COVID-19 (β_5_)	–0.0324	.18		

^a^AR: first-order autocorrelation maximum likelihood estimate.

^b^D-W: Durbin-Watson test.

### The Proportion of Patients Receiving Opioid Prescriptions From Multiple Providers

The proportion of patients receiving opioid prescriptions from multiple providers was found to be the lowest in the pre-NIMS period (172/24,924, 0.69%), followed by the post-NIMS pre–COVID-19 period (160/15,642, 1.02%), and then the highest in the post–COVID-19 period (69/5016, 1.37%; Table 1). A sudden increase in the level was seen after the introduction of NIMS (β_2_=.6252; *P*=.004). A slight increase was observed after the COVID-19 outbreak, which was not significant (β_4_=.3969; *P*=.09). No significant change in the trend of patients receiving opioid prescriptions from multiple providers was observed after the implementation of NIMS or the COVID-19 outbreak. The segmented regression results of the trend and level changes are displayed in Table 2 and Figure 2B.

### The Overlap Rate of Opioid Prescriptions Per Patient

The overlap rate of opioid prescriptions per patient showed a slight increase, with the lowest in the pre-NIMS period (486/24,924, 1.95%), followed by the post-NIMS pre–COVID-19 period (339/15,642, 2.17%), and then the highest in the post–COVID-19 period (102/5016, 2.30%; Table 1).

Both the NIMS and COVID-19 did not appear to significantly impact the level and trend of the overlap rate of opioid prescriptions per patient (Table 2). The trend and level changes are illustrated in Figure 2C.

### The Naloxone Use Rate Among Opioid Users

The naloxone use rate among opioid users declined over time, from 0.57% (142/24,924) in the pre-NIMS period, to 0.51% (80/15,642) after NIMS, and then further down to 0.45% (23/5016) in the post–COVID-19 period (Table 1).

The baseline trend (β_1_) before NIMS introduction was .0156 (*P*=.01), which was turned down by –0.0117 to 0.0039 after NIMS. COVID-19 changed little of the trend (*P*=.18). Abrupt decline was observed after the introduction of NIMS (β_2_=–.2968; *P*=.04). The regression results are shown in Table 2 and Figure 2D.

## Discussion

### Overview

Our study aimed to assess the impact of the Korean health care IT for narcotics management, known as the NIMS, on opioid misuse using nationally representative claims data. The study findings indicate that the NIMS did not demonstrate significant improvement during the 32 months since its introduction in the 4 specific indicators we selected. Rather, we observed a gradual increase in high-dose opioid prescribing and an abrupt increase in multiple providers’ prescriptions following the implementation of NIMS. While these changes were statistically significant, their clinical significance appears to be relatively minor, warranting further investigation.

The use rate of naloxone, which serves as a proxy for opioid misuse or abuse, had been increasing over time before the implementation of NIMS, and a temporary decrease in the use rate of naloxone was observed after NIMS implementation. This decrease suggests that the NIMS may have had a positive impact on opioid-related outcomes by reducing the occurrence of opioid misuse or abuse. However, it is inconclusive because a concurrent increase in the number of opioid users during the same period may have also contributed to the decrease in naloxone use rate.

We also conducted a second analysis, not treating COVID-19 as an interruption, and found no significant differences. While the primary focus of our study is to assess the impact of NIMS, this additional analysis confirms that even when not considering COVID-19 as an interruption, the NIMS still did not show a significant impact on the identified indicators of opioid overuse and misuse.

Given the limited existing research in this area, the reasons for NIMS not yielding anticipated results remain largely unexplored. In this regard, some recent studies have raised workforce concerns [36]. A recent study reported that the workforce to operate the NIMS is not adequately secured. Essential tasks such as analysis of causes of misuse and corrective actions for detected problems were carried out only to a limited extent due to the lack of workforce reinforcement [36]. Furthermore, recent in-depth interviews with 3 prescribers and 2 pharmacists confirmed that health care professionals primarily use the NIMS to fulfill legal reporting requirements [37]. These latest studies have raised the need for the introduction of a more comprehensive measure such as “opioid stewardship,” which encompasses guidelines for health care professionals, education for patients and the public, and enhanced communication and coordination among key stakeholders [38,39].

In comparison to similar systems overseas, the lack of real-time access to patient’s prescription histories within the NIMS may explain its suboptimal impact on drug usage enhancement [21]. The United States has a state-based electronic database called prescription drug monitoring programs (PDMPs), and some Canadian provinces operate the narcotics monitoring system (NMS), which mandates that pharmacies submit dispensing information for all monitored drugs [21]. NIMS, PDMPs, and NMS share the common goal of monitoring and managing the prescription and dispensing of narcotics to address misuse and abuse [21]. While they share this overarching objective, these systems differ in their operations and functionalities compared to the NIMS. All 3 systems collect, monitor, and analyze prescription data for controlled substances, including opioids. However, a key distinction is that PDMPs and NMS provide real-time access to patients’ prescription histories for prescribers and pharmacists, enabling them to promptly identify potential misuse, overuse, or diversion of prescription medications [40]. Numerous studies have investigated the effectiveness of PDMPs and NMS, with some demonstrating a significant reduction in the prescription of narcotic drugs and a reduction in opioids misuse [41-44]. These findings underscore the pivotal role of real-time monitoring in curbing prescription abuse and misuse.

The study population had a higher representation of women than men, who are known to experience more severe and long-lasting pain, leading to a higher prescription rate for opioids [45]. Additionally, it is noteworthy that our study population included a higher proportion of MedAid beneficiaries (ranging from 8.8% to 10.7%) compared to the general population, where the percentage of MedAid beneficiaries typically remains around 3% [46]. Several factors may contribute to the higher presentation of MedAid beneficiaries in our study. Research has shown that this population often experiences higher rates of chronic pain [47,48] and may have limited access to alternative pain management treatments [49,50]. Moreover, one important aspect to consider is the distinction in copayment requirements between NHI and MedAid. Under the NHI system, patients are required to make copayments for health care services, including medications such as opioids. On the other hand, MedAid beneficiaries generally pay lower or no copayments for medications, including opioids. This distinction can lead to different use patterns, a phenomenon often referred to as “moral hazard” [26,51,52]. While our study primarily focused on assessing the impact of NIMS on opioid use, the observed variation in MedAid beneficiaries’ opioid use patterns highlight an avenue for further research.

To the best of our knowledge, this research is the first study to directly evaluate the impact of the NIMS on opioid use using nationwide real-world data, providing invaluable insights into its effect on opioid prescription patterns in South Korea and suggesting areas for improvement. Nevertheless, our study has several limitations that should be noted. First, the inclusion of patients with musculoskeletal and connective tissue disorders may limit the generalizability of the findings to other patients with different medical conditions. Furthermore, the study’s setting in South Korea may limit the generalizability of the findings to other health care systems and countries with different opioid prescription practices and regulatory frameworks. Variations in health care policies, cultural factors, and clinical guidelines may influence opioid use patterns in distinct ways, warranting caution when extrapolating the study’s conclusions to other regions. Second, data analyzed in this study are based on reimbursement claims, meaning that any opioids prescribed outside of the national insurance market would not be recorded. This could result in an underrepresentation of actual opioid prescription quantities [53]. Third, the diagnostic codes assigned to patients may not always be completely accurate, which could impact the accuracy of the findings. Additionally, being a quasi-experimental study using ITS, there might be uncontrolled confounding factors that could influence the observed trends in opioid use.

It is worth noting that the NIMS has undergone improvements. For instance, the “Information Network to Prevent Doctor Shopping for Narcotics” feature, similar to PDMPs, included the entire narcotics data set since March 2021. This integration allows prescribers to assess a patient’s history of medical narcotic use, particularly when a doctor shopping for narcotics is suspected [20,21]. Feedback reports grounded in the NIMS big data are sent to doctors who demonstrate problematic prescribing behavior (eg, prescribing substantial amounts of medical narcotics). This series of changes is expected to gradually materialize in the form of opioid stewardship mentioned above in South Korea. Given these circumstances, there arises a clear need for future investigations to assess the efficacy of the enhanced NIMS.

### Conclusion

This study suggests that, in its current form, the NIMS may not have brought significant improvements to the identified indicators of opioid overuse and misuse. Additionally, the COVID-19 outbreak exhibited no significant influence on opioid use patterns. The absence of a real-time monitoring feature within the NIMS could be a key contributing factor. Further exploration and enhancements are needed to maximize the NIMS’ impact on curbing inappropriate opioid use.

## Appendix

Multimedia Appendix 1 Disease and medication codes.

## Abbreviations

HIRA: Health Insurance Review and Assessment Service
HIRA-NPS: Health Insurance Review and Assessment Service–National Patient Sample
ICD-10: International Classification of Diseases, Tenth Revision
ITS: interrupted time series
MedAid: Medical Aid
MFDS: Ministry of Food and Drug Safety
MME: morphine milligram equivalents
NHI: National Health Insurance
NIMS: Narcotics Information Management System
NMS: narcotics monitoring system
PDMP: prescription drug monitoring program
PVI: Patriots and Veterans Insurance Plan

## Data Availability

The authors used the Health Insurance Review and Assessment Service–National Inpatient Sample data for this study and do not have permission to share these data. Raw data can be accessed with permission from the Health Insurance Review and Assessment.
