# Supplementary material for: Impact of the Narcotics Information Management System on Opioid Use Among Outpatients With Musculoskeletal and Connective Tissue Disorders: Quasi-Experimental Study Using Interrupted Time Series
Source: JMIR Public Health Surveill. 2024 Feb 21;10:e47130. doi: 10.2196/47130 (PMC10918548; doi:10.2196/47130)
Supplement: Multimedia Appendix 1 [file publichealth_v10i1e47130_app1.docx]

Table S1. Disease and medication codes.

| Disease | Codes |
| --- | --- |
| Arthropathies | M00-M25 |
| Dorsopathies | M40-M54 |
| Soft tissue disorders | M60-M79 |
| Other disorders of the musculoskeletal system and connective tissue | M30-M36, M95-M99 |
| Medication |  |
| Codeine | 137703AT, 313400AC |
| Dihydrocodeine | 144901AT |
| Fentanyl | 158209CP, 158210CP, 158211CP, 158212CP, 158213CP, 158216CP |
| hydrocodone | 518200AT, 518300AT |
| Hydromorphone | 441102AT, 441103AT, 441106AT, 441107AT, 441108AT, 441109AT, 441110AT |
| Morphine | 197301AT, 197302AT, 197305AT |
| Oxycodone | 359001AT, 359002AT, 359003AT, 359004AT, 359007AT, 359008AT, 380500AT, 517100AT, 517200AT, 564000AT, 564100AT, 667600AT |
| Tapentadol | 628401AT, 628402AT, 628403AT, 628404AT |
